# 4D flow for accurate assessment of differential pulmonary arterial flow in patients with tetralogy of Fallot

**DOI:** 10.1186/1532-429X-11-S1-O52

**Published:** 2009-01-28

**Authors:** Alison K Meadows, Michael D Hope, David Saloner, Charles B Higgins

**Affiliations:** University of California San Francisco, San Francisco, CA USA

**Keywords:** Main Pulmonary Artery, Branch Pulmonary Artery, Single Acquisition, Ventricular Stroke Volume, Gradient Echo Pulse Sequence

## Objective

To demonstrate that 4D flow, in a single acquisition, can provide efficient, accurate, and complete data sets to determine differential pulmonary blood flow in patients with Tetralogy of Fallot (TOF).

## Background

Patients with tetralogy of Fallot (TOF) often have branch pulmonary artery (PA) stenoses and abnormalities in flow distribution. Such abnormalities can be addressed and have an impact on clinical management. Distorted anatomy, turbulent flow jets, and/or metallic stents often make the choice of 2D phase contrast flow planes difficult, time consuming, and labor intensive. As a result, studies may be completed without collection of the data necessary to determine flow distribution. 4D flow techniques permit the collection of temporally-resolved 3D data sets of the central systemic and pulmonary vasculature in a single acquisition. Complete data acquisition is guaranteed and appropriate planes for flow quantification can be chosen during post processing.

## Methods

Employed was a temporally-resolved, 3D phase contrast technique (4D flow), optimized for blood flow analysis in the thoracic vasculature. Data was acquired using an RF-spoiled 3D gradient echo pulse sequence with velocity encoding in 3 spatial directions. All measurements were performed on a 1.5 T clinical scanner (Signa CV/I, GE, Milwaukee, WI) using an 8-channel cardiac coil. Scan parameters were as follows: VENC = 160–200 cm/s; fractional FOV = 300 × 270 mm^2^, slab thickness = 78 mm, and matrix = 256 × 192 × 30 yielding a spatial resolution of 1.17 × 1.56 × 2.60 mm^3^. Within each cardiac cycle, the in-plane phase encode value was held constant while 4 slice-encoding phase encodes are acquired to encode all flow directions. Parallel imaging (GRAPPA) with an acceleration factor of 2 was used. Scan times ranged from 12–16 minutes depending on heart rate. Retrospective EKG gating was used to resolve 20 time frames through the cardiac cycle yielding a temporal resolution of 50–80 msec. Respiratory compensation was employed. The raw data was reconfigured for EnSight visualization (CEI Inc., Apex, NC). Navigation within the 3D data set allows retrospective placement of planes perpendicular to the vessel of interest. 10 subjects with TOF were prospectively enrolled. For each subject, a 4D flow data set was obtained and an attempt was made to obtain 2D flow in 4 locations; the ascending aorta (AsAo), main pulmonary artery (MPA), and branch PA's (RPA and LPA).

## Results

**Figure 1:**
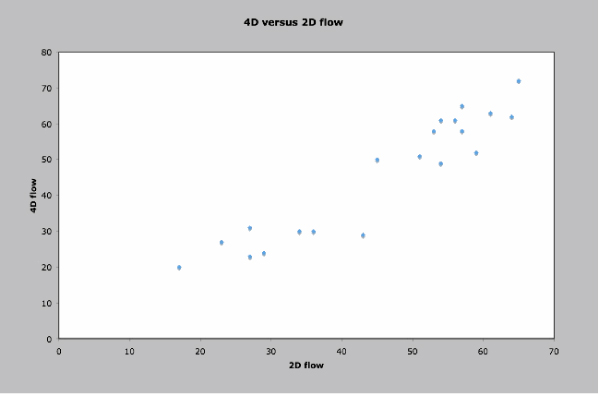
**4D versus 2D flow**.

**Figure 2:**
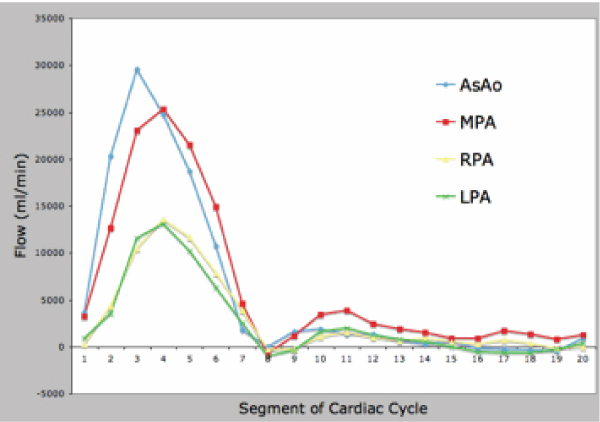
**Healthy subject pulmonary flow**.

**Figure 3:**
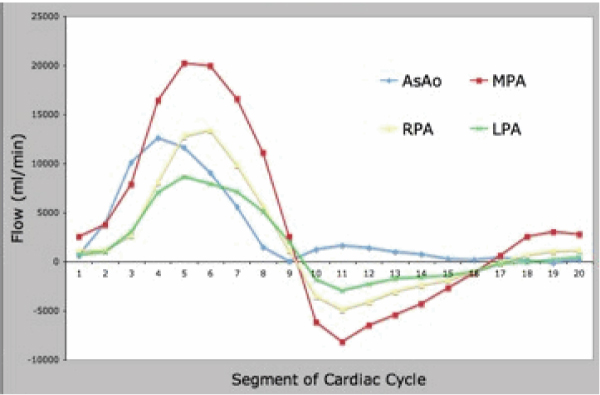
**Flow profile**.

Of a total of 40 data points, there were 16 missing 2D data points and 7 missing 4D data points. Missing 2D data were secondary to aliasing and inappropriate prescription of 2D flow planes. Missing 4D data were secondary to aliasing. There were 20 total paired data points (Figure 1). There was good correlation between the two techniques (Spearman's rho value of 0.91, p < 0.0001). The 4D flow data were internally consistent: AsAo flow equaled left ventricular stroke volume, MPA flow equaled right ventricular stroke volume, and the sum of the branch PA flows equaled MPA flows, all to within 10%. This internal consistency is demonstrated in Figures 2 and 3, which display flows in the AsAo, MPA, RPA, and LPA over the cardiac cycle in a normal subject and a patient with TOF respectively. Figure 4 and 5 present streamlines in the pulmonary arteries at peak systole in the axial and sagittal planes respectively.

**Figure:**
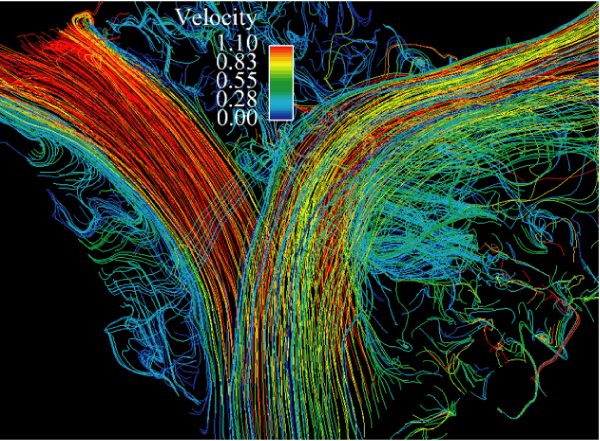
Figure 4

**Figure:**
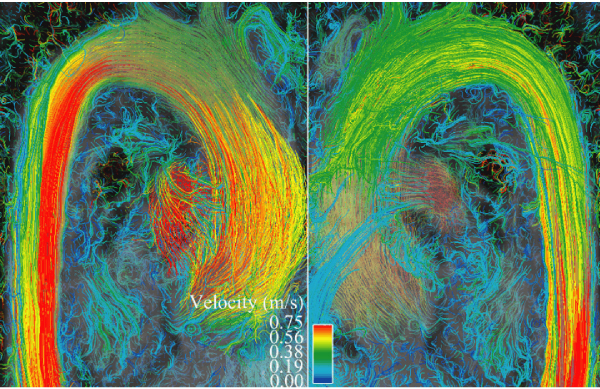
Figure 5

## Conclusion

4D flow provides complete and accurate assessment of differential pulmonary blood flow in patients with Tetralogy of Fallot (TOF) in a single acquisition. Good correlation between 4D and 2D techniques for blood flow quantification in the central systemic and pulmonary vasculature is demonstrated. Navigation within 4D flow data sets allows placement of planes at will without being hindered by the prospective prescription of traditional 2D flow techniques.

